# Comparative accuracy of oro-helix length, naso-tragus length, and gestational age-based methods for endotracheal tube depth prediction in neonates ≤1,500 g

**DOI:** 10.3389/fped.2026.1861978

**Published:** 2026-07-27

**Authors:** Stergiani Agorastos, Wei Hou, Echezona T. Maduekwe

**Affiliations:** 1 Department of Pediatrics, Neonatology, Stony Brook Children’s Hospital, Stony Brook, NY, United States; 2 Family, Population and Preventive Medicine, Stony Brook University Hospital, Stony Brook, NY, United States

**Keywords:** endotracheal intubation, gestational age, malposition, neonatal intensive care, neonates

## Abstract

**Objective:**

To evaluate the accuracy and reliability of the Oro-helix length (OHL), Naso-tragus length (NTL), and gestational age-based table (GA table) in predicting the ideal endotracheal tube (ETT) depth in neonates.

**Study design:**

This prospective study enrolled orally intubated neonates weighing ≤1,500 g admitted to a level IIIC neonatal intensive care unit (NICU) from April 2022 to February 2024. For each patient, the ETT insertion depths were predicted using OHL and NTL measurements, along with the GA table. To compare the accuracy of these methods, all predicted depths were assessed against the ideal depth determined by chest radiograph (CXR). Statistical analyses were conducted using a linear regression Bland–Altman model.

**Results:**

Fifty were enrolled with a mean gestational age of 26.6 ± 1.5 weeks and a mean intubation weight of 953 ± 246.8 g. Among the three methods compared, NTL measurements demonstrated the greatest bias (−0.61 cm) and less agreement (−1.78 to 0.56 cm) than either OHL (−0.96 to 1.27 cm) or the GA table (−1.37 to 0.91 cm). OHL had the least bias (0.15 cm) and showed the strongest correlation with gestational age (slope = 0.09, R2 = 0.12, p = 0.02). In contrast, the correlations with gestational age for NTL and the GA table were weaker and not statistically significant (NTL: slope = 0.05, 95% CI: −0.02 to 0.13, R2 = 0.04, *p* = 0.16; GA table: slope = 0.02, 95% CI: −0.04 to 0.07, R2 = 0.01, *p* = 0.58).

**Conclusion:**

In neonates ≤1,500 g, OHL is the most accurate and reliable method for predicting ETT depth. OHL outperforms the NTL and GA tables based on CXR and thus may potentially be preferred approach for accurate ETT depth determination.

## Introduction

Endotracheal tube (ETT) intubation is a critical, life-saving procedure frequently performed in the neonatal intensive care unit (NICU) to secure and maintain the airway of newborn infants. This intervention is particularly essential for very low birth weight infants who are at increased risk of respiratory failure. While ETT placement is routinely performed in neonatology, incorrect tube positioning is also frequently encountered. Malposition can lead to a spectrum of complications, including inadequate ventilation, trauma to the larynx, lung collapse (atelectasis), accidental removal of the tube if it is placed too high at the thoracic inlet, or more severe outcomes such as pneumothorax and interstitial emphysema if the tube is advanced too far, particularly into the right main bronchus (1–3). To minimize these risks, it is crucial to confirm that the tip of the ETT is ideally positioned. The optimal location for the ETT tip, as visualized on a chest radiograph (CXR), is midway between the clavicles and the carina. In neonates, this corresponds anatomically to the space between the first (T1) and second (T2) thoracic vertebral bodies on the CXR, which serves as the reference for correct ETT placement (4).

Various approaches have been proposed to simplify and improve the accuracy of endotracheal tube (ETT) insertion depth estimation in neonates. Clinicians traditionally used the weight-based 7-8-9 rule from Tochen's study, which linked ETT depth in centimeters to infant weight in kilograms (5). However, later studies showed this method often resulted in overly deep ETT placement, especially in very low birth weight infants (1, 6–8). To address these concerns, the Neonatal Resuscitation Program (developed by the American Academy of Pediatrics and the American Heart Association) now recommends two alternatives for determining the optimal ETT depth. These methods include using the NTL and GA tables (9).

Craniofacial proportions vary among ethnic groups, so adding a fixed number—like 1 cm, to estimate ETT depth may not always be accurate. The NTL method, a distance between the base of the nasal septum and the tip of the tragus on either side of the face, plus 1 cm, was introduced by Shukla et al. as a guide to ETT depth (10). However, a study by Tzu-Chiang et al. found considerable discrepancy in all Asian infants. In these cases, the optimal depth is often deferred (based on the weight) from the estimated provided by the NTL (11). Thus, these ethnicity-specific anatomical factors challenge the universal applicability of the NTL method. On the other hand, Flinn et al., in a randomized study, did not find a significant difference in the ETT malposition rate between the 7-8-9 rule and the gestation-based guideline (12), and, as a result, raised questions about the superiority of the GA table over the replaced 7-8-9 rule.

Our previous study showed that OHL prediction of ideal ETT depth was more reliable than the 7-8-9 rule in infants ≤1,500 g (13). This study compares the OHL, NTL, and GA table methods for predicting ETT tip position, as measured against CXR, in infants ≤1,500 g. We hypothesize that OHL will more accurately predict optimal ETT depth than NTL and GA tables in these infants.

## Materials and methods

This was a prospective observational study approved by the Institutional Review Board and conducted from April 2022 through February 2024 at Stony Brook Children's Hospital (Stony Brook, New York), a level IIIC Neonatal Intensive Care Unit. All orally intubated neonates were enrolled in the study within 48 h of admission into the NICU and the parents/guardians of eligible patients gave written informed consent prior to enrollment in the study. Neonates with craniofacial or vertebral anomalies, or whose parents declined consent, were excluded from the study.

Infants were intubated at the discretion of the primary care team, and the NRP intubation guidelines were followed. CXRs were taken with the infant's head in a neutral position per standard unit practice, and the inserted ETT depths were adjusted to acceptable locations as per the reading by the pediatric radiologist, who was unaware of the infant's involvement in the study. The ETTs were secured using a Neo-BarÒ (Neotech Products, California, USA) in a midline position, with an identifiable insertion depth at the upper lip.

Using the initial post-intubation CXR, we compared three insertion depths to the ideal ETT depth: the NTL, GA table, and the OHL depth. The ideal ETT depth was defined as the area between the first and second thoracic vertebrae as seen on CXR. The NTL and the OHL were measured on the left side of the enrolled infant from the nasal septum to the tragus and from the angle of the mouth to the ear helix, respectively, by two independent medical providers who were blinded to the ideal ETT depth and to each other, using a measuring tape marked in centimeters. The number “1” was added to the measured NTL value to obtain the predicted ETT depth. The left side was arbitrarily chosen, as our previous study showed no significant difference between left- and right-sided OHL in infants ≤1,500 g (13). For consistency, NTL measurements were also performed on the left side. Maternal and neonatal demographic data were collected from the electronic medical records.

### Study outcomes

Our primary outcome was to evaluate how closely the predicted endotracheal tube (ETT) insertion depths matched the ideal ETT depth for each patient. Predictions were made using the OHL, NTL, and GA table methods. We also explored several secondary outcomes. These included the reproducibility of OHL measurements between independent observers and the frequency with which each method resulted in the ETT being placed in the optimal position. We defined suboptimal position if the ETT depth position was >0.5 cm above the upper border of first thoracic vertebra or below the lower border of second thoracic vertebra. In addition, we assessed whether gestational age affected the accuracy of the ETT depth prediction methods by evaluating the correlation between gestational age and the predicted ETT depths relative to the ideal ETT depths.

### Statistical analysis

*A priori* sample size calculation determined that 50 neonates were required for a paired *t*-test to detect a mean difference of 0.5 cm with 99% power at a 5% significance level, assuming a standard deviation of 0.79 cm.

CXR was used as the standard to determine the ideal ETT depth. Statistical analyses were done using SAS version 9.4 (SAS Institute, Cary, North Carolina), and results were considered significant if *P* < 0.05. For each prediction method, the mean and standard deviation of the differences from the ideal ETT depth were calculated to assess accuracy. Inter-rater reliability of the OHL measurements between the two independent providers was assessed using the Intraclass Correlation Coefficient (ICC), based on a two-way random-effects model for absolute agreement. Also, paired mean differences were used to compare the three methods. Simple linear regression compared each prediction method with gestational age. Agreement between prediction methods and the ideal ETT depth was checked using Bland–Altman analysis, where the “difference” was defined as CXR minus the predicted ETT depths. Instead of the ±2 cm margin used in previous study (14), we used a ±1 cm margin for greater safety and to lower the risk of incorrect ETT placement. Bland–Altman limits of agreement with 95% confidence intervals were set as ±1.96 times the standard deviation of the bias.

## Results

**Figure 1:**
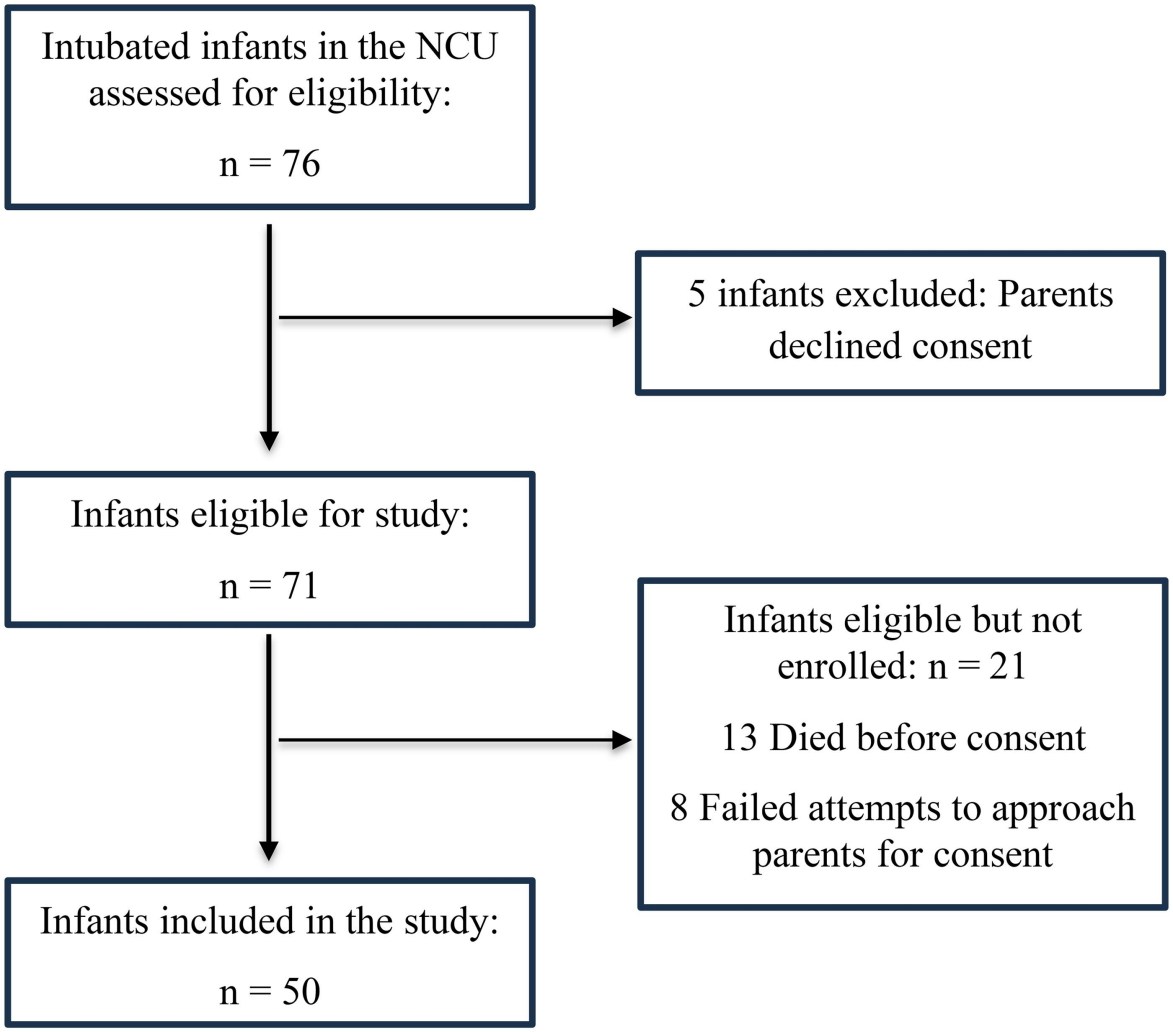
Flow diagram of study subjects.

During the study period, a total of 76 neonates were evaluated for eligibility, and 50 were successfully enrolled, (see Figure 1). The demographic data of the cohort is detailed in Table 1. After the evaluation of the baseline characteristics, most of the infants (84%) were delivered through cesarean section.

**Table 1:** Demographic characteristics of study infants (*n* = 50).

Independent factors	Level	Number	Percentage
Gender	F	25	50
	M	25	50
Gestational age, wks. (Mean ± SD)	26.6 ± 1.5	50	100
Birth weight, gram (Mean ± SD)	953.2 ± 246.8	50	100
Race	Caucasian	24	48
	Black	8	16
	Hispanic	5	10
	Asian	2	4
	Other	11	22
Mode delivery	Vaginal	8	16
	C-section	42	84

F, female; M, male; C-section, cesarean section; SD, standard deviation

### Comparing the ETT depth predicted by OHL, NTL, and GA table to the CXR ideal depth

The OHL predicted ETT depths were not significantly different from the ideal CXR depth; the mean (±SD) predicted ETT depth was 6.06 ± 0.64 cm versus 6.22 ± 0.62 cm, with a mean difference of 0.15 ± 0.57 cm (95% CI, −0.01 to 0.32; *p* = 0.06). By contrast, NTL predicted ETT depths differed significantly from the ideal CXR depths; the mean predicted ETT depth was 6.83 ± 0.62 cm versus 6.22 ± 0.62 cm, with a mean difference of 0.61 ± 0.59 cm (95% CI, 0.44–0.78; *p* < 0.001). The GA table predicted mean ETT depth was also significantly different from the ideal CXR depth: 6.42 ± 0.46 cm versus 6.22 ± 0.62 cm, with a mean difference of −0.2 ± 0.59 cm (95% CI, −0.37 to −0.03; *p* = 0.02), as shown in Table 2.

**Table 2:** Comparison of the ETT depth predicted by the OHL, NTL, GA table and the ideal CXR ETT depth.

Variable	Min (cm)	Max (cm)	Mean	SD	Mean Diff	95% CI	*p*-value
OHL ETT depth prediction (*n* = 50)							
OHL ETT depth	4.30	7.40	6.06	0.64	0.15	−0.01 to 0.32	0.06
Ideal CXR depth	5.10	7.70	6.22	0.62			
NTL ETT depth prediction (*n* = 50)							
NTL ETT depth	4.40	7.80	6.83	0.62	−0.61	−0.78 to −0.44	<0.001
Ideal CXR depth	5.10	7.70	6.22	0.62			
GA Table ETT depth prediction (*n* = 50)							
GA Table ETT depth	5.50	7.00	6.42	0.46	−0.20	−0.37 to 0.03	0.02
Ideal CXR depth	5.10	7.70	6.22	0.62			

OHL predicted ETT depths were not significantly different from the ideal CXR depth. Min, minimum value; Max, maximum value; SD, standard deviation; Mean Diff, mean difference; 95% CI, 95% confidence interval; OHL, oro-helix length; CXR, chest-x-ray; NTL, naso-tragus length; GA Table, gestational age-based table.

**Figure 2:**
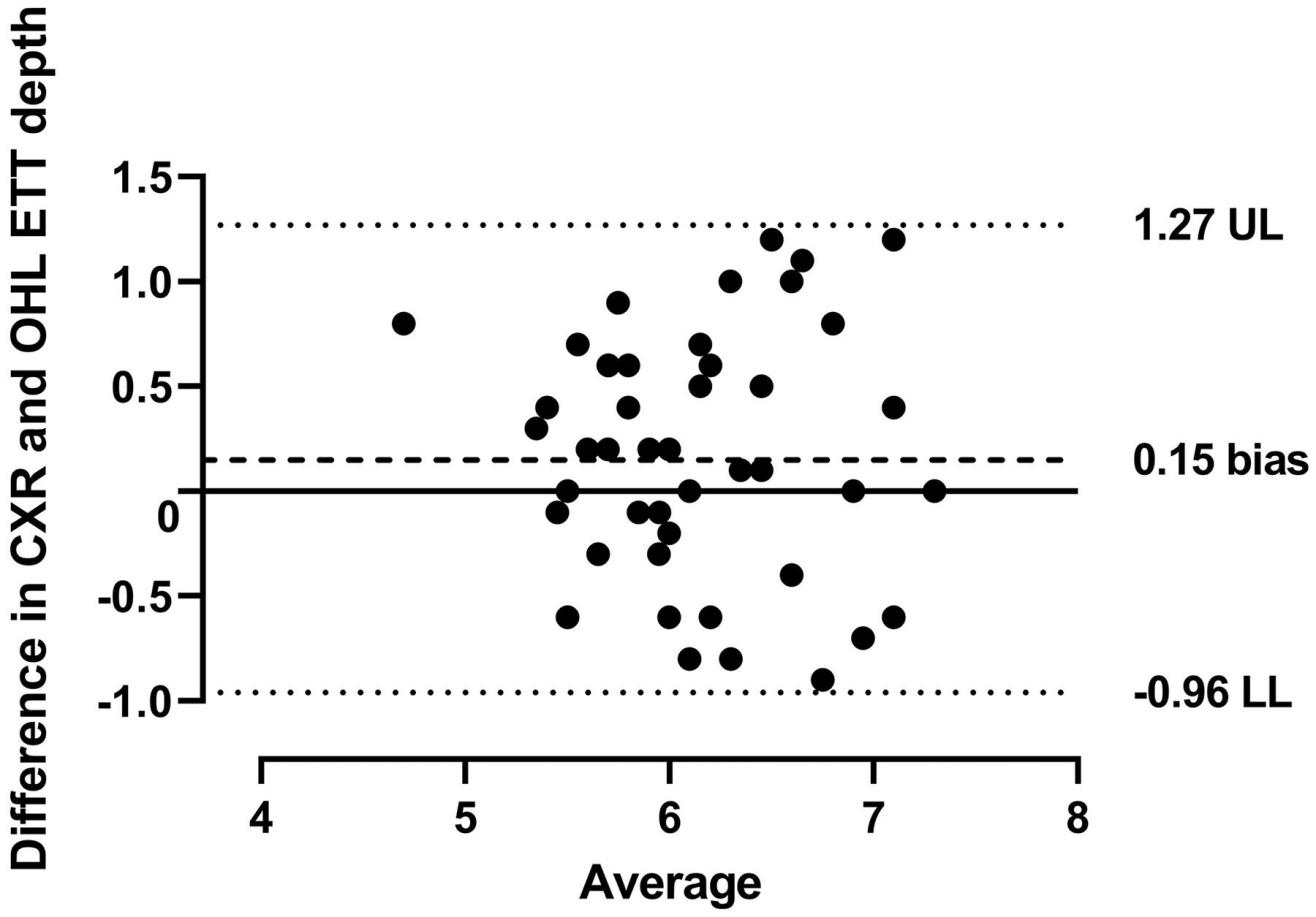
Comparing OHL predicted ETT depths to CXR ideal depths. The Bland–Altman plot shows agreement between CXR and OHL ETT depth using linear regression and a 95% confidence interval. The solid line represents perfect agreement, while the dotted lines indicate the limits of agreement at ±1.96 SD. UL, upper limit; LL, lower limit; CXR, chest-x-ray; OHL, oro-helix length; ETT, endotracheal tube.

**Figure 3:**
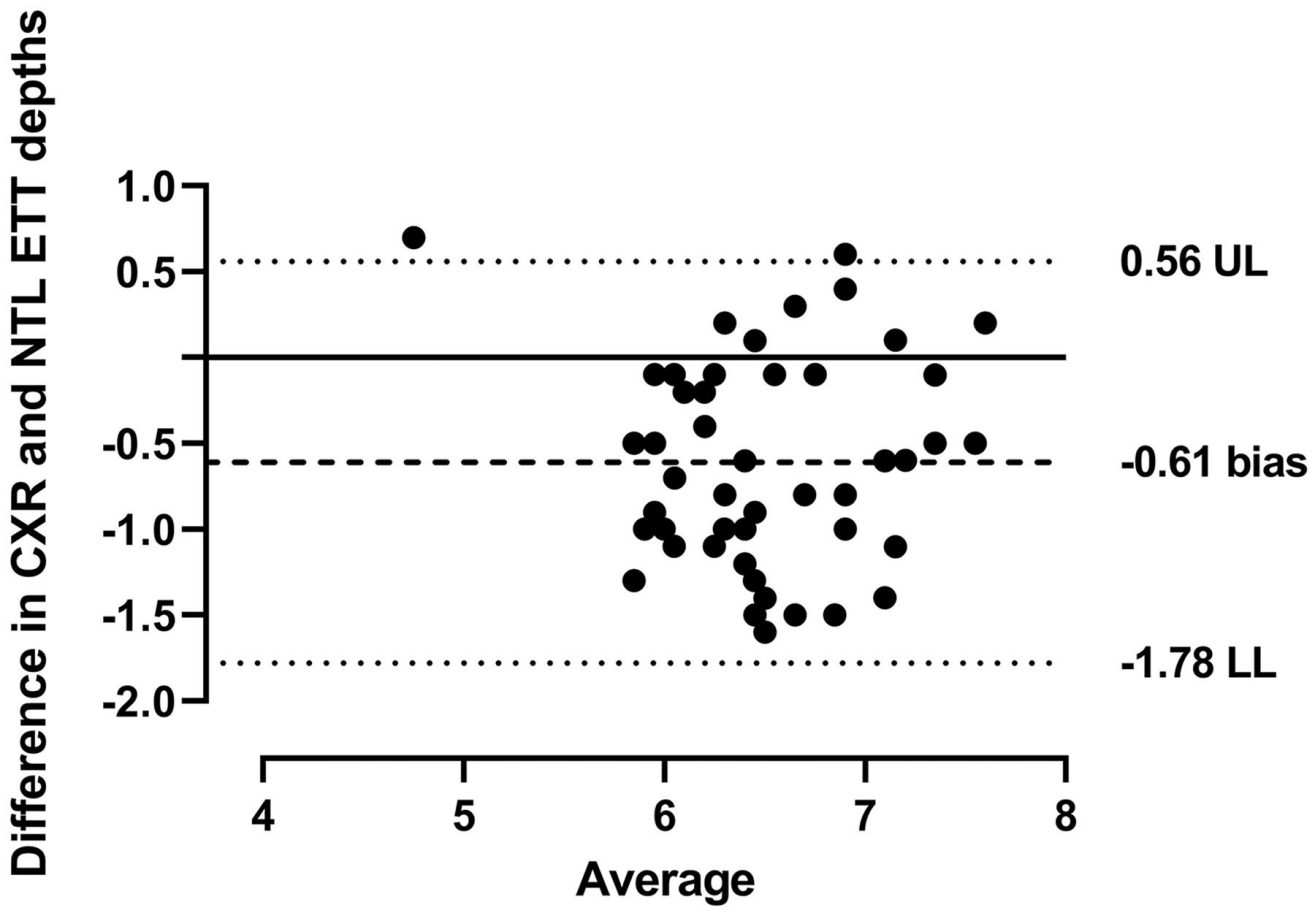
Comparing NTL predicted ETT depths to CXR ideal depths. The Bland–Altman plot shows agreement between CXR and NTL ETT depths using linear regression and a 95% confidence interval. The solid line represents a perfect agreement, while the dotted lines indicate the limits of agreement at ±1.96 SD. UL, upper limit; LL, lower limit; CXR, chest-x-ray; NTL, naso-tragus length; ETT, endotracheal tube.

**Figure 4:**
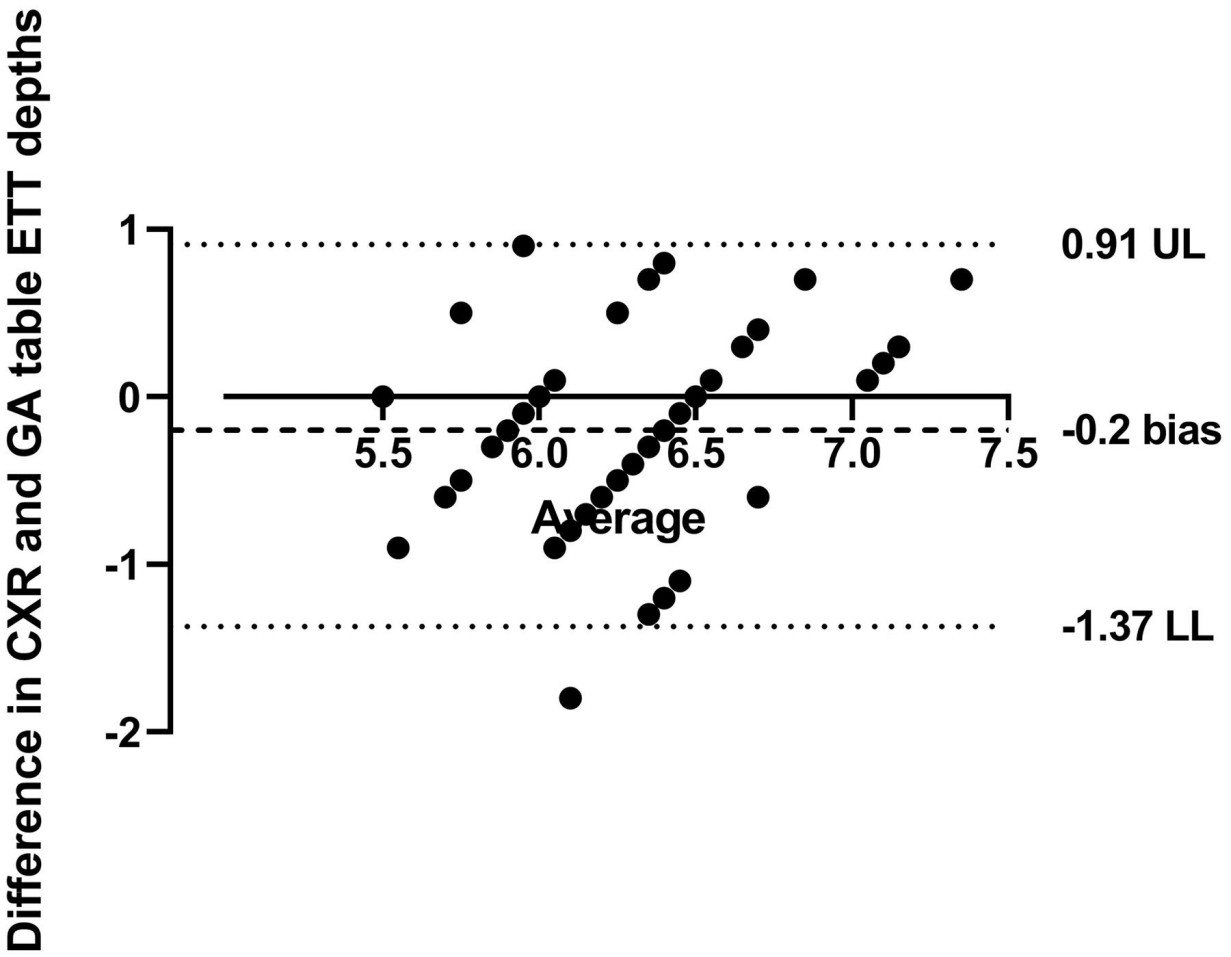
Comparing GA table predicted ETT depths to CXR ideal depths. The Bland–Altman plot shows agreement between CXR and GA table ETT depths using linear regression and a 95% confidence interval. The solid line represents a perfect agreement, while the dotted lines indicate the limits of agreement at ±1.96 SD. UL, upper limit; LL, lower limit; CXR, chest-x-ray; GA, gestational age; ETT, endotracheal tube.

The Bland–Altman analysis highlights the mean bias between predicted and ideal CXR ETT depths: 0.15 cm for OHL, −0.61 cm for NTL, and −0.2 cm for the GA table. The lower limits of agreement (LL) compared to ideal CXR depths were −0.96 for OHL, −1.78 for NTL, and −1.37 for the GA table. The upper limits of agreement (UL) were 1.27 for OHL, 0.56 for NTL, and 0.91 for the GA table, as shown in Figures 2–4.

### Comparing the reproducibility of OHL and the proportion of predicted ETT depth by OHL, NTL, and GA table in ideal position

We found that the reproducibility of the OHL measurements across the 50 pairs using ICC (2,1) was 0.99 [95% CL (0.98, 1.00)]. In addition, OHL method, compared to the NTL and GA table, yielded the highest level of agreement with CXR-confirmed ideal positioning, with 95% of its predictions (46/50) <0.5 cm from either the upper border of T1 or lower border of T2, compared to the GA table method that correctly predicted 40 of 50 ETT depths (90%) and the NTL method with 74% correct predictions (37/50).

### Correlation between gestational age and predicted ETT depth relative to the CXR ideal depths

**Figure 5:**
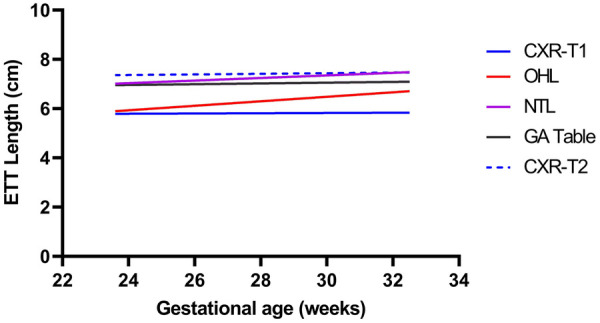
Correlation between the ETT depth prediction methods and the gestational age. OHL demonstrates the strongest positive correlation with gestational age. CXR, chest-x-ray; T1, first thoracic vertebra; T2, second thoracic vertebra; OHL, oro-helix length; NTL, naso-tragus length; GA, gestational age; cm, centimeters.

We found that all three ETT depth prediction methods were linearly related to gestational age. However, the OHL method demonstrated the highest proportion of variation that could be explained by gestational age (slope = 0.09, 95% CI: 0.02–0.16, R2 = 0.12, *p* = 0.02) when compared to the NTL method (slope = 0.05, 95% CI: −0.02 to 0.13, R2 = 0.04, *p* = 0.16) and the GA table method (slope = 0.02, 95% CI: −0.04 to 0.07, R2 = 0.01, *p* = 0.58) as shown in Figure 5 and Table 3.

**Table 3:** Correlation summary between the ETT depth prediction methods and gestational age.

Variable	Slope	95% CI	R squared	*P* value
OHL	0.0911	0.02 to 0.16	0.12	0.02
NTL	0.0523	−0.02 to 0.13	0.04	0.16
GA Table	0.0155	−0.04 to 0.07	0.01	0.58

OHL demonstrates a highest R squared value with gestational age. OHL, oro-helix length; NTL, naso-tragus length; GA, gestational age; CI, confidence interval.

## Discussion

In clinical settings, there is ongoing discussion about the best way to predict and guide optimal ETT depth during neonatal intubation, with many practitioners relying on the NTL and GA tables as recommended by the NRP (9). In our prospective study comparing the OHL method with these established approaches in 50 neonates, we observed that the OHL method is highly reliable as an ETT depth predictor in neonates weighing ≤1,500 g at birth. This finding aligns with our previous study (13). Additionally, the OHL method is a more accurate optimal ETT depth predictor than either the NTL or the GA table methods, a finding that could influence future practice. However, while most values predicted by the OHL method fell within the ideal ETT depth range (between T1 and T2), there may be a tendency for the OHL predicted depth to slightly underestimate the optimal CXR ETT depth, suggesting that although the OHL and CXR ETT depths are generally comparable, confirmatory radiological imaging remains an important safety step. These findings support the idea that the OHL method's greater predictive accuracy could make it the preferred approach for predicting optimal ETT placement in neonates weighing ≤1,500 g, especially when accuracy is paramount, and CXR confirmation is not immediately available.

This study is the first prospective study comparing the OHL method with the NTL and GA table methods for ETT depth prediction in neonates admitted to the NICU with birthweights ≤1,500 g. The choice of ideal ETT depth prediction method remains controversial because the evidence for replacing the traditional weight-based formula (adding 6 cm to an infant's weight in kilograms) with the NTL or GA table methods is limited. In our study, we found that predicted ETT depth using NTL consistently differed from the CXR depth measurements (*p* < 0.001), with a mean overestimation of −0.6 cm. This suggests that the reliance on the NTL method may lead to consistent overestimation of actual ETT depth, potentially leading to inadvertent endobronchial intubation. Similarly, there was a statistically significant difference (*p* = 0.02) between the GA table predicted and CXR measured ETT depth, with the GA table predicting a deeper ETT depth than was observed on CXR. The mean difference, while relatively small (−0.2 cm), may have clinical implications, as small deviations in ETT placement in this patient population can result in malposition.

In addition, there is a reasonable agreement between the ideal CXR ETT depths and those predicted by the NTL and GA table in the Bland–Altman evaluation, while the NTL has a mean bias of −0.61 cm with limit of agreement of −1.78 cm (LL) to 0.56 cm (UL), the GA table has a mean bias of −0.2 cm and limit of agreement of −1.37 cm (LL) to 0.91 cm (UL). This demonstrates that while the NTL shows greater variability and tends to overestimate the ideal ETT depth by >0.5 cm, the GA table shows moderate variability and is slightly deeper than the ideal CXR ETT depth. In addition, the percentage of the predicted ETT depth in the ideal position differed: 74% with the NTL method and 90% with the GA table method. While these trends in the overestimation of the ideal ETT depth by the NTL and GA table methods are consistent with previous studies (12, 15, 16), the percentage in ideal position we observed with the GA table (90%) was inconsistent with the 70% reported by Priyadarshi et al. (16). This observed discrepancy may stem from variations in study populations, unintentional tube migration during fixation, or excessive care by clinicians seeking to minimize extubation risks. In contrast, our findings demonstrate a small mean difference between the predicted and optimal CXR dept (0.15 cm), which was not statistically significant. This finding suggests that the OHL method provides ETT depth estimates that are closely aligned with those determined radiographically, which is consistent with our previous study (13). The limits of agreement of −0.96 cm (LL) to 1.27 cm (UL), suggest most predictions fall within about ±1 cm of the ideal. In addition, the OHL method achieved a percentage in ideal position of 95%, which was slightly higher than that in our previous study (13). This difference could be explained by the smaller cohort of neonates weighing ≤1,500 g in our previous study. Our findings, therefore, suggest that while the OHL and GA table ETT depth predictions are closer to the ideal CXR depth, the OHL with the narrowest range and smallest mean bias aligns most closely with the ideal CXR depth among the three methods.

Our results on reproducibility of the OHL measurements demonstrate an exceptionally high degree of agreement, as evidenced by the ICC of 0.99, indicating that the OHL measurements are highly consistent between observers. This level of consistency is critical in both clinical and research settings, as it ensures that measurements are not influenced by the individual conducting the assessment. The high ICC we observed may be attributable to both the providers' experience and the clear landmark for the OHL measurement. Future work could investigate the reliability of these measurements with less experienced observers. Nonetheless, the current results underscore the reliability of OHL measurements when performed by individuals using the specified landmarks.

Furthermore, we observed that all three ETT depth prediction methods demonstrated a modest linear relationship with gestational age, but the strength of this relationship was limited. The OHL method yielded the highest R^2^ value (0.12, *p* = 0.02), meaning that gestational age accounted for only 12% of the variance in predicted ETT depth. Although this association reached statistical significance, the low R^2^ value indicates that gestational age is a poor predictor of optimal ETT depth across these prediction methods.

This study has notable strengths. It is the first prospective analysis to directly compare the OHL, NTL, and GA table methods for predicting ETT depth in neonates ≤1,500 g, providing valuable new evidence in this area. The prospective design increases reliability by reducing retrospective bias. The study offers practical insights by comparing the accuracy rates of all three methods, thus helping clinicians optimize ETT placement when a CXR is not immediately available.

Despite these strengths, the study has several limitations. First, its single-institution design may introduce bias from unique local practices and protocols, thus limiting generalizability. Therefore, multi-center research is needed to confirm findings across diverse clinical environments. Second, our study's sample of 50 neonates weighing ≤1,500 g may not reflect the broader neonatal population, especially regarding racial and demographic diversity. Larger, more inclusive studies could address this limitation. Third, 11 out of our 50 enrolled subject (22%), identified as “other” without specifying their race. This limits our ability to interpret subgroup differences by race and thus avoid misrepresenting clinically relevant differences. An attempt in future study to collect data with clear definition of race will minimize this ambiguity and increase completeness. Fourth, while bedside CXR and ultrasound are both used to determine ETT depth in neonates, we used only CXR as our gold standard. Although CXR interpretation may vary among interpreters, ultrasound's reliability also depends on the sonographer's proficiency. For our study, ultrasonographic determination of the ETT depth in neonate was not the standard of care at our institution at the time. Finally, prediction accuracy was evaluated solely by the ideal ETT depth on CXR, which may differ from the actual clinical placement due to ETT manipulation and fixation techniques. Prospective multicenter studies are needed to clarify how clinical practices affect the reliability of ETT depth prediction in neonates.

## Conclusion

In conclusion, our study demonstrates that the OHL method provides a more accurate prediction of optimal ETT depth in neonates weighing ≤1,500 g compared to NTL and GA table methods. This increased accuracy suggests that the OHL method may be preferred in a clinical setting where accurate ETT placement is critical and an immediate CXR is unavailable. However, the study's imitations underscore the need for larger, multicenter studies to validate these findings and assess applicability across diverse neonatal populations.

## Data Availability

The original contributions presented in the study are included in the article/Supplementary Material, further inquiries can be directed to the corresponding author.
